# Cotton host resistance as a tool for managing *Rotylenchulus reniformis* in Louisiana

**DOI:** 10.2478/jofnem-2024-0014

**Published:** 2024-04-22

**Authors:** Tristan T. Watson

**Affiliations:** LSU AgCenter, Department of Plant Pathology and Crop Physiology, 302 Life Science Building, Baton Rouge, Louisiana, 70803, United States

**Keywords:** Upland cotton, *Gossypium hirsutum*, reniform nematode, *Rotylenchulus reniformis*, Louisiana, host resistance

## Abstract

The reniform nematode, *Rotylenchulus reniformis*, is a major yield-limiting pest of upland cotton (*Gossypium hirsutum*) in the United States that has been steadily increasing in incidence in many states. Reniform nematode-resistant cotton cultivars have recently become commercially available for cotton producers; however, few field trials have evaluated their efficacy as a nematode management tool. The aim of this study was to evaluate reniform nematode population development, plant growth, and seed cotton yield of reniform nematode-resistant cotton cultivars in two nematode-infested fields in Louisiana. Replicated small-plot field trials were conducted in St. Joseph, LA (NERS field) and Winnsboro, LA (MRRS field) during the 2022 and 2023 growing seasons. In 2022, cultivars evaluated included: (1) DP 1646 B2XF (susceptible/tolerant), (2) DP 2141NR B3XF (resistant), (3) PHY 332 W3FE (resistant), (4) PHY 411 W3FE (resistant), and (5) PHY 443 W3FE (resistant). In 2023, an additional susceptible cotton cultivar, PHY 340 W3FE, was also included. All nematode-resistant cotton cultivars evaluated provided suppression of reniform nematode population development relative to that of the susceptible cotton cultivars, with suppression of nematode soil population densities at harvest ranging from 49 – 81% relative to DP 1646 B2XF. The resistant cultivar PHY 411 W3FE provided the most consistent suppression of reniform nematode population development, reducing reniform nematode soil population densities at harvest in both field locations and both trial years. In contrast, DP 2141NR B3XF only reduced soil population densities at harvest in the NERS field in 2023. Despite relatively consistent nematode suppression and improvements in plant vigor ratings and canopy coverage associated with the resistant cotton cultivars, a yield increase was only observed with PHY 332 W3FE and PHY 411 W3FE planted at the NERS field in 2023. Despite strong resistance to reniform nematode in the evaluated cotton cultivars, nematode soil population densities still increased during the growing season in plots planted with resistant cotton cultivars, emphasizing the need for additional management tactics to use alongside host resistance. This study indicates that new reniform nematode-resistant cotton cultivars show promising potential to reduce nematode population development during the growing season in Louisiana.

The reniform nematode, *Rotylenchulus reniformis* Linford & Oliveira, is a major yield-limiting pest of upland cotton (*Gossypium hirsutum* L.) in the Southeastern United States (Koenning et al. 2004). The reniform nematode was first documented in 1940 on cowpea in Hawaii (Linford and Oliveira 1940) and was subsequently reported a year later on cotton in Louisiana (Smith and Taylor 1941). Feeding by this semi-endoparasitic nematode often leads to stunting of plant growth, reduced boll number and size, and decreased lint quality (Jones et al. 1959). Heavily infested fields often show uniformly stunted plant growth across the entire field, making diagnosis difficult without collecting soil samples for nematode extraction and quantification (Birchfield and Jones 1961). In Louisiana, the incidence of reniform nematode in cotton production fields has been steadily increasing since the 1980s (Robinson 2007). Recently conducted disease loss estimates in Louisiana predict that reniform nematode is responsible for a 1.5% yield loss in the state, corresponding to approximately 4,200 bales of cotton lost to this nematode species (Lawrence et al. 2022). In addition to the damage caused to cotton, many growers in Louisiana often rotate cotton fields with susceptible soybean varieties, emphasizing the need for effective management strategies for this damaging pest.

Given the increasing prevalence of reniform nematode in Louisiana production fields and a crop rotation scheme that often includes rotating between two highly susceptible hosts, there is a great need for effective nematode management practices. Effective management of any plant-parasitic nematode often requires an integrated approach, and reniform nematode on cotton is no exception. Numerous nematicides have been registered for use on cotton over the years, and the application of these products to reniform nematode-infested fields has often improved cotton lint yields (Birchfield 1963; Lawrence and McLean 1999; McLean and Lawrence 2003; Koenning et al. 2007). Unfortunately, the majority of these nematicides only provide temporary suppression of reniform nematode population development during the early stages of crop establishment, with late season nematode population development commonplace in nematicide-treated soils (Lawrence and McLean 1999; Koenning et al. 2007). Crop rotation to a non-host, such as corn and grain sorghum, can also be used for management of reniform nematode populations in a field (Davis et al. 2003); however, such rotations may not be as economically profitable as cropping with a susceptible crop, such as cotton and soybean. Cotton host resistance has been used to reduce damage associated with other nematode species, including southern root-knot nematode (*Meloidogyne incognita*) (Ogallo et al. 1997; Koenning et al. 2001; Wheeler et al. 2014). Deploying effective cotton host resistance to reniform nematode may provide an additional management tool for reniform nematode in cotton production. (Turner et al. 2023).

Until recently, no cotton cultivars with effective resistance to reniform nematode were commercially available to producers. The search for reniform nematode resistance in cotton started in the 1960s when 24 cotton cultivars were screened for reniform nematode resistance in a greenhouse study; however, all cultivars were found to be susceptible (Birchfield and Brister 1963). Since this initial study, over 2,000 upland cotton germplasm lines have been evaluated against reniform nematode, and none have been found to be highly resistant (Yik and Birchfield 1984; Robinson and Percival 1997; Robinson et al. 2004; Weaver et al. 2007). Given the lack of apparent reniform nematode resistance in upland cotton, research efforts switched to focusing on introgressing reniform nematode resistance from other *Gossypium* species (Yik and Birchfield 1984). Screening of *Gossypium* species has indicated that *Gossypium barbadense* is highly resistant to reniform nematode (92% suppression) (Yik and Birchfield 1984); however, the subsequently developed hybrid breeding line, BARBREN-713, only provided moderate reniform nematode suppression, and the level of suppression varied by growing region (Bell et al. 2015). Research efforts then switched to introgressing reniform resistance from *Gossypium longicalyx*, which has been shown to support no reniform nematode reproduction in preliminary screening work (Bell and Robinson 2004). A triple species hybrid (*G. hirsutum* x *G. longicalyx* × *G. armourianum*) was developed and backcrossed with upland cotton cultivars to develop the LONREN breeding lines with high resistance to reniform nematode (Bell et al. 2014). Unfortunately, when the LONREN breeding lines were challenged with a high inoculum level of reniform nematode, a hypersensitive response reaction led to reduced root mass, which ultimately stunted the plants (Sikkens et al. 2011). Quantitative trait loci associated with reniform nematode resistance in BARBREN-713 (*Ren^Barb2^*) and LONREN-1 (*Ren^lon^*) have since been identified and utilized in molecular-assisted cotton breeding programs (Nichols et al. 2010; Wubben et al. 2017). In 2021, the first commercially available cotton cultivars with stacked resistance to reniform and southern root-knot nematode were released under the tradenames Deltapine^®^ DP 2141NR B3XF, PhytoGen^®^ PHY 332 W3FE, and PhytoGen^®^ PHY 443 W3FE. In 2022, an additional resistant cultivar was released as PhytoGen^®^ PHY 411 W3FE.

The utility of new reniform-nematode-resistant cotton cultivars for nematode suppression is largely unknown. In a recent field study conducted in Alabama, the resistant cotton cultivar PHY 332 W3FE reduced reniform nematode egg production and increased yield relative to the susceptible cultivar PHY 340 W3FE (Turner et al. 2023). Preliminary microplot studies in Louisiana suggest that DP 2141NR B3XF can suppress reniform nematode population development and improve yield relative to the susceptible cultivar DP 1646 B2XF (Watson 2022); however, more studies are needed with new germplasm releases. The aim of this study was to evaluate reniform nematode population development, plant growth, and seed cotton yield of newly available reniform-nematode-resistant cotton cultivars in two nematode-infested fields in Louisiana.

## Materials and Methods

### Site descriptions

In 2022 and 2023, cotton fields were planted at two locations in Louisiana to evaluate the utility of new cotton cultivars with resistance to reniform nematode. The first field was located at the LSU AgCenter Northeast Research Station (NERS) in St. Joseph, Louisiana, United States (31.942155779069132, −91.22663016858824). The soil at the site is a Bruin silt loam (49% sand, 42% silt, 9% clay, 0.8% organic matter, 5.8 pH, 4.8 meq/100 g cation exchange capacity). The site has a history of moderate reniform nematode infestation, and the trial was planted in a location that was planted with soybean in the prior growing season. The second field was located at the LSU AgCenter Macon Ridge Research Station (MRRS) in Winnsboro, Louisiana, United States (32.13687481246169, −91.69727009027044). The soil at the site is a Gigger silt loam (35% sand, 48% silt, 17% clay, 1.3% organic matter, 5.4 pH, 6.3 meq/100 g cation exchange capacity). The site has a history of severe reniform nematode infestation and was also planted with soybean in the prior growing season. For both field locations, irrigation, fertilization, insecticide, and fungicide applications were followed according to commercial production practices for Louisiana.

### Experimental Design and Measurements

At both field locations, the experimental design was a randomized complete block with five replications of each cultivar. In 2022, cultivars included: (1) Deltapine^®^ DP 1646 B2XF (susceptible), (2) Deltapine^®^ DP 2141NR B3XF (resistant), (3) PhytoGen^®^ PHY 332 W3FE (resistant), (4) PhytoGen^®^ PHY 411 W3FE (resistant), and (5) PhytoGen^®^ PHY 443 W3FE (resistant). In 2023, an additional susceptible cotton cultivar, PhytoGen^®^ PHY 340 W3FE, was also included. Cultivars were grown as small plots that were 10.7 m long by four rows wide. Rows were spaced 96.5 cm apart at NERS and 101.6 cm apart at MRRS. The trial was planted on May 19, 2022, and May 18, 2023, at the NERS field and on May 17, 2022, and May 23, 2023, at the MRRS field using a 420 seeds/plot planting density.

Stand count was measured at 14 and 28 days after planting (DAP). On each measurement date, the number of plants within a row was counted in four randomly selected 1.52-m subsections from within the center two rows of each plot. Data are presented as the number of plants per hectare. Plant vigor was measured at 56 DAP. Within each replicate, the most vigorous plot was assigned a value of 100%, and all subsequent plots within the replicate were assigned a vigor rating relative to the most vigorous plot. Data are presented as percent vigor.

Canopy coverage was measured at 56 DAP. Images were collected on a Samsung Galaxy S21 smartphone (Samsung Electronics, Suwon-si, South Korea) using a 1X zoom at a height of 1.52 m above the row bed and default image acquisition settings. Images were collected from four randomly selected locations within the center, two rows from each plot. Images were analyzed for canopy coverage using Canopeo software with vegetation type set to cotton (Patrignani and Ochsner 2015). Data are presented as percent canopy coverage.

The population density of soil nematodes was determined at planting, mid-season (56 DAP), and at harvest. Twelve randomly selected soil cores (20 – 25 cm in depth, 2.5 cm in diameter) were obtained from the center two rows of each plot. When plants were present (mid-season and harvest sampling dates), soil cores were removed approximately 5 cm from the stem. Soil cores from each plot were thoroughly mixed, then placed into a plastic bag and stored at 12 °C for a maximum of 14 days prior to subsequent processing. Nematodes were extracted from a 250-cm^3^ subsample of soil from each plot using a semi-automatic elutriator (Byrd et al. 1976) combined with the sugar flotation technique (Jenkins 1964). After collecting the nematodes over a 25-μM sieve, the nematode samples were transferred in water into plastic vials and stored at 12 °C for a maximum of two weeks prior to counting. The abundance of reniform nematode was determined using an inverted compound microscope. Data are presented as number of nematodes per 500 cm^3^ of soil.

Seed cotton was harvested on October 20, 2022, and October 13, 2023, at the NERS field and on October 2, 2022, and October 18, 2023, at the MRRS field. Yield data was collected from the center two rows of each plot using a two-row cotton picker. Data are presented as kilograms of seed cotton per hectare.

### Statistical analysis

Data from the field trials were subjected to a one-way ANOVA in SAS OnDemand for Academics (SAS Institute Inc. 2014, Cary, NC) using the Mixed Model procedure. The experimental model was a randomized complete block with five blocks as random effects and cotton cultivars as fixed effects. Differences in treatment means were examined using Tukey’s HSD test (*P* < 0.05).

## Results

### 2022 Growing Season

At the NERS field in 2022, seedling establishment did not differ among the five cotton cultivars at either measurement date (Table 1). Mid-season plant vigor ratings were greater for three of the resistant cotton cultivars, PHY 332 W3FE, PHY 411 W3FE, and PHY 443 W3FE, than DP 1646 B2XF. The cultivars PHY 332 W3FE and PHY 443 W3FE also improved crop vigor relative to DP 2141NR B3XF. Canopy coverage showed a similar trend to that of plant vigor, with all three of the resistant PhytoGen^®^ cultivars (PHY 332 W3FE, PHY 411 W3FE, and PHY 443 W3FE) displaying increased canopy coverage relative to that of DP 1646 B2XF. Despite differences in vigor and canopy coverage, no differences in seed cotton yield were observed among the five cotton cultivars, which yielded 2,899 – 3,256 kilograms of seed cotton per hectare at the NERS field. Reniform nematode soil population densities at the NERS field in 2022 averaged 5,472 – 8,832 nematodes per 500 cm^3^ of soil at the time of planting the field (Table 2). By the mid-season sampling date, reniform nematode soil population densities in the plots planted with DP 2141NR B3XF, PHY 411 W3FE, and PHY 443 W3FE were reduced by 39.3 – 56.8 % relative to that of plots planted with DP 1646 B2XF. At harvest, only PHY 411 W3FE and PHY 443 W3FE continued to maintain lower reniform nematode soil population densities relative to DP 1646 B2XF, resulting in 57.5 % and 54.9 % fewer nematodes in soil by the end of the growing season, respectively.

**Table 1:** Stand count, crop vigor, canopy coverage, and seed cotton yield of cotton cultivars planted at the NERS field in 2022. DAP refers to days after planting. Values sharing the same letter within a column do not differ significantly (*P*-value>0.05).

Cultivar	Stand Count (#/ha)		Vigor (%)		Canopy Coverage (%)		Seed Cotton Yield (kg/ha)

	14 DAP	28 DAP					
DP 1646 B2XF	92,457	67,302	68	c	22	b	3,192
DP 2141NR B3XF	108,091	95,175	75	bc	35	ab	2,899
PHY 332 W3FE	105,373	89,736	96	a	42	a	2,979
PHY 411 W3FE	99,255	89,057	88	ab	41	a	3,168
PHY 443 W3FE	105,373	98,575	95	a	41	a	3,256

*P*-value	0.714	0.164	<0.001		0.001		0.861

**Table 2:** Soil population densities of reniform nematode (*Rotylenchulus reniformis*) at planting, mid-season, and harvest at the NERS field in 2022. Values sharing the same letter within a column do not differ significantly (*P*-value>0.05).

Cultivar	*R. reniformis* / 500 cm^3^ soil				

	At Plant	Mid-Season		Harvest	
DP 1646 B2XF	5,472	13,328	a	15,744	a
DP 2141NR B3XF	6,112	5,762	b	11,128	ab
PHY 332 W3FE	8,000	9,056	ab	9,088	ab
PHY 411 W3FE	8,832	8,096	b	6,688	b
PHY 443 W3FE	7,392	7,072	b	7,104	b

*P*-value	0.583	0.047		0.015	

At the MRRS field in 2022, seedling establishment did not differ among the five cotton cultivars at either of the two measurement dates (Table 3). All four resistant cotton cultivars improved mid-season plant vigor ratings relative to that of DP 1646 B2XF; however, PHY 332 W3FE and PHY 411 also had higher plant vigor ratings than DP 2141NR B3XF. Canopy coverage was greater in the PHY 411 W3FE and PHY 443 W3FE plots than in plots planted with DP 1646 B2XF. No differences in seed cotton yield were observed among the five cotton cultivars, which yielded between 1,469 and 1,551 kilograms of seed cotton per hectare at the MRRS field. Reniform nematode soil population densities at the MRRS field in 2022 averaged 17,152 – 25,952 nematodes per 500 cm^3^ of soil at the time of planting the field (Table 4). By the mid-season sampling date, reniform nematode soil population densities in the plots planted with DP 2141NR B3XF, PHY 332 W3FE, and PHY 411 W3FE were reduced by 38.9 – 48.6 % relative to that of plots planted with DP 1646 B2XF. At harvest, only PHY 411 W3FE continued to maintain lower reniform nematode soil population densities relative to DP 1646 B2XF, resulting in 70.3% fewer nematodes in soil by the end of the growing season.

**Table 3:** Stand count, crop vigor, canopy coverage, and seed cotton yield of cotton cultivars planted at the MRRS field in 2022. DAP refers to days after planting. Values sharing the same letter within a column do not differ significantly (*P*-value>0.05).

Cultivar	Stand Count (#/ha)		Vigor (%)		Canopy Coverage (%)		Seed Cotton Yield (kg/ha)

	14 DAP	28 DAP					
DP 1646 B2XF	65,263	67,302	76	c	30	b	1,551
DP 2141NR B3XF	67,984	67,984	84	b	35	ab	1,469
PHY 332 W3FE	90,416	87,018	98	a	39	ab	1,534
PHY 411 W3FE	77,500	79,541	97	a	41	a	1,528
PHY 443 W3FE	64,583	60,504	91	ab	42	a	1,578

*P*-value	0.160	0.171	<0.001		0.022		0.993

**Table 4:** Soil population densities of reniform nematode (*Rotylenchulus reniformis*) at planting, mid-season, and harvest at the MRRS field in 2022. Values sharing the same letter within a column do not differ significantly (*P*-value>0.05).

Cultivar	*R. reniformis* / 500 cm^3^ soil				

	At Plant	Mid-Season		Harvest	
DP 1646 B2XF	20,384	23,232	a	47,616	a
DP 2141NR B3XF	17,152	13,200	b	27,776	ab
PHY 332 W3FE	17,408	12,416	b	22,016	ab
PHY 411 W3FE	25,952	14,184	b	14,144	b
PHY 443 W3FE	24,160	18,176	ab	26,304	ab

*P*-value	0.293	0.044		0.048	

### 2023 Growing Season

At the NERS field in 2023, seedling establishment did not differ among the six cotton cultivars at either measurement date (Table 5). Mid-season plant vigor ratings were greater for PHY 332 W3FE than the susceptible cultivar PHY 340 W3FE. Canopy coverage showed a similar trend, with PHY 332 W3FE displaying increased canopy coverage relative to that of PHY 340 W3FE and DP 1646 B2XF. Seed cotton yield was greatest with PHY 332 W3FE, which averaged 1,522 – 1,905 more kilograms per hectare of seed cotton relative to that of DP 1646 B2XF, DP 2141NR B3XF, PHY 340 W3FE, or PHY 443 W3FE. The cotton cultivar PHY 411 W3FE yielded 1,141 more kilograms per hectare of seed cotton than DP 2141NR B3XF and 1,265 more kilograms per hectare of seed cotton than PHY 340 W3FE. Reniform nematode soil population densities at the NERS field in 2023 averaged 320 – 2,624 nematodes per 500 cm^3^ of soil at the time of planting the field (Table 6). By the mid-season sampling date, reniform nematode soil population densities in the plots planted with either of the four resistant cotton cultivars were lower than plots planted with DP 1646 B2XF (59.6 – 70.1 % reduction) or PHY 340 W3FE (62.8 – 72.3 % reduction). At harvest, PHY 332 W3FE, PHY 411 W3FE, and PHY 443 W3FE continued to maintain lower reniform nematode soil population densities relative to DP 1646 B2XF or PHY 340 W3FE.

**Table 5:** Stand count, crop vigor, canopy coverage, and seed cotton yield of cotton cultivars planted at the NERS field in 2023. DAP refers to days after planting. Values sharing the same letter within a column do not differ significantly (*P*-value>0.05).

Cultivar	Stand Count (#/ha)		Vigor (%)		Canopy Coverage (%)		Seed Cotton Yield (kg/ha)	

	14 DAP	28 DAP						
DP 1646 B2XF	48,947	61,865	81	ab	60	b	2,743	bc
DP 2141NR B3XF	61,865	54,385	75	ab	78	ab	2,484	c
PHY 340 W3FE	43,508	46,906	70	b	64	b	2,360	c
PHY 332 W3FE	55,744	70,022	98	a	87	a	4,265	a
PHY 411 W3FE	53,706	62,545	76	ab	71	ab	3,625	ab
PHY 443 W3FE	41,469	45,549	78	ab	74	ab	2,743	bc

*P*-value	0.435	0.071	0.022		0.031		<0.001	

**Table 6:** Soil population densities of reniform nematode (*Rotylenchulus reniformis*) at planting, mid-season, and harvest at the NERS field in 2023. Values sharing the same letter within a column do not differ significantly (*P*-value>0.05).

Cultivar	R. reniformis / 500 cm^3^ soil				

	At Plant	Mid-Season		Harvest	
DP 1646 B2XF	704	10,624	a	77,696	a
DP 2141NR B3XF	2,624	3,520	b	39,940	bc
PHY 340 W3FE	2,560	11,520	a	73,216	ab
PHY 332 W3FE	2,432	3,200	b	31,488	c
PHY 411 W3FE	1,088	3,840	b	15,168	c
PHY 443 W3FE	320	4,288	b	20,736	c

*P*-value	0.218	<0.001		<0.001	

At the MRRS field in 2023, DP 2141NR B3XF had 31,271 and 30,592 more established seedlings per hectare at 14 DAP relative to that of PHY 340 W3FE and PHY 443 W3FE (Table 7); however, no differences in seedling establishment were observed by 28 DAP. All four resistant cotton cultivars improved mid-season plant vigor ratings relative to that of PHY 340 W3FE; however, only DP 2141NR B3XF, PHY 411 W3FE, and PHY 443 W3FE had higher plant vigor ratings relative to that of DP 1646 B2XF. Canopy coverage was greater in the PHY 332 W3FE and PHY 443 W3FE plots relative to that of plots planted with DP 1646 B2XF, DP 2141NR B3XF, or PHY 340 W3FE. No differences in seed cotton yield were observed among the six cotton cultivars, which yielded between 3,537 and 3,982 kilograms of seed cotton per hectare at the MRRS field. Reniform nematode soil population densities at the MRRS field in 2023 averaged 3,520 – 5,504 nematodes per 500 cm^3^ of soil at the time of planting the field (Table 8). No differences were observed in soil population densities at the mid-season sampling date. At harvest, reniform nematode soil population densities were lower in plots planted with PHY 332 W3FE and PHY 411 W3FE relative to that of plots planted with DP 1646 B2XF or PHY 340 W3FE.

**Table 7:** Stand count, crop vigor, canopy coverage, and seed cotton yield of cotton cultivars planted at the MRRS field in 2023. DAP refers to days after planting. Values sharing the same letter within a column do not differ significantly (*P*-value>0.05).

Cultivar	Stand Count (#/ha)			Vigor (%)		Canopy Coverage (%)		Seed Cotton Yield (kg/ha)

	14 DAP		28 DAP					
DP 1646 B2XF	60,504	ab	67,302	70	bc	29	c	3,879
DP 2141NR B3XF	72,061	a	81,579	94	a	35	bc	3,982
PHY 340 W3FE	40,790	b	57,785	66	c	32	bc	3,646
PHY 332 W3FE	63,901	ab	64,578	83	ab	44	a	3,578
PHY 411 W3FE	66,622	ab	70,701	90	a	40	ab	3,938
PHY 443 W3FE	41,469	b	81,577	92	a	45	a	3,537

*P-value*	0.008		0.148	<0.001		<0.001		0.517

**Table 8:** Soil population densities of reniform nematode (*Rotylenchulus reniformis*) at planting, mid-season, and harvest at the MRRS field in 2023. Values sharing the same letter within a column do not differ significantly (*P*-value>0.05).

Cultivar	*R. reniformis* / 500 cm^3^ soil			

	At Plant	Mid-Season	Harvest	
DP 1646 B2XF	4,480	8,576	95,740	a
DP 2141NR B3XF	5,504	4,160	66,896	ab
PHY 340 W3FE	4,096	6,016	87,984	a
PHY 332 W3FE	3,520	6,464	38,724	b
PHY 411 W3FE	4,672	3,200	45,696	b
PHY 443 W3FE	4,160	5,760	68,752	ab

*P*-value	0.924	0.786	0.048	

## Discussion

The recent release of cotton cultivars resistant to reniform nematodes provides growers with a valuable new tool for nematode management in cotton production in the United States. In this study, all nematode-resistant cotton cultivars evaluated provided suppression of reniform nematode population development relative to that of susceptible cotton cultivars, with suppression of soil population densities at harvest ranging from 49 – 81% relative to the susceptible Deltapine^®^ cultivar (DP 1646 B2XF). In a recently conducted study in Alabama, field trials demonstrated that reniform nematode egg production on the resistant cultivar PHY 332 W3FE was 80% lower than the susceptible cultivar PHY 340 W3FE (Turner et al. 2023). Similar levels of reduction in reniform nematode egg production have been observed on other cotton germplasm lines with reniform nematode resistance when screened in other field trials conducted in Alabama (Bell et al. 2023). In the current study, the resistant cultivar PHY 411 W3FE provided the most consistent suppression of reniform nematode soil population densities, whereas the resistant cultivar DP 2141NR B3XF only reduced soil population densities at harvest in one field trial. These data provided additional support for initial reports of only partial resistance to reniform nematode in the resistant Deltapine^®^ cotton cultivar (Turner et al. 2023). All nematode-resistant cotton cultivars evaluated in this study supported some level of reniform nematode population development during the growing season, indicating that host resistance should be considered as an additional tool to be used alongside other management tactics in an integrated management approach.

Despite the relatively consistent suppression of reniform nematode on the resistant cotton cultivars in this study, a yield benefit was only observed on select resistant cultivars in one growing season and at one field location. This is in contrast with recent studies conducted in Alabama, which demonstrated greater cotton lint yields on resistant cotton cultivars relative to susceptible cultivars when planted into reniform-nematode-infested fields (Bell et al. 2023; Turner et al. 2023). The apparent lack of yield response in the current study is unlikely to be related to differences in local weather conditions among the field sites, as similar amounts of precipitation occurred at both fields during each growing season (NERS received 64 cm of precipitation in 2022 and 35 cm in 2023; MRRS received 73 cm of precipitation in 2022 and 31 cm in 2023); however, the addition of water stress during the 2023 growing season may have contributed to the yield response observed at the NERS field relative to the 2022 growing season. In the current study, many of the resistant cotton cultivars had higher plant vigor ratings and greater canopy coverage when compared to susceptible cotton cultivars, suggesting that increases in plant size did not necessarily correspond to increases in seed cotton yield. The apparent lack of yield increase in resistant cotton cultivars relative to DP 1646 B2XF may be related to the high tolerance that this cotton cultivar has to reniform nematode feeding (Grabau 2022), but this does not explain the lack in yield response relative to the highly susceptible cultivar PHY 340 W3FE (Turner et al. 2023). Moreover, the current study did not assess differences in lint yield or quality, which may have been negatively impacted in the susceptible cotton cultivars (Jones et al. 1959).

This study evaluated the utility of new reniform nematode-resistant cotton cultivars in two growing seasons and at two field locations in Louisiana, allowing for the evaluation of cultivar performance under varying levels of nematode pressure. Reniform nematode pressure was greater at the MRRS field relative to the NERS field during both growing seasons, and pressure was greater in the 2022 growing season relative to the 2023 season. A yield increase associated with planting resistant cotton cultivars was only observed at the NERS field in 2023, where initial reniform nematode soil population densities were low (<3,000 nematodes per 500 cm^3^ soil). This may suggest that resistant cotton cultivars perform better in fields with low reniform nematode pressure. This is in contrast to a recent study conducted in Alabama, where a reniform nematode-resistant cotton cultivar (PHY 332 W3FE) outyielded the susceptible cotton cultivar (PHY 340 W3FE) at varying levels of nematode pressure observed among trial years (Turner et al. 2023). Differences in cotton cultivar performance among growing regions in the United States may be related to environmental differences as well as potentially differences in the virulence of the endemic reniform nematode populations (Khanal et al. 2018), emphasizing the need for additional trials with reniform nematode-resistant cotton cultivars throughout representative production areas of the United States.

In Louisiana, the nematode-resistant cotton cultivar PHY 411 W3FE showed the most consistent suppression of reniform nematode soil population densities; however, all resistant cultivars suppressed nematode population development to some extent. Even with the lack of apparent yield benefit associated with growing a resistant cotton cultivar, incorporating resistant cultivars could still be beneficial in reducing reniform nematode pressure in rotation schemes utilizing other susceptible crops, such as soybean (Kularathna et al. 2019). Despite strong resistance to reniform nematode in the evaluated cotton cultivars, nematode soil population densities still increased during the growing season in plots planted with resistant cotton cultivars, emphasizing the need for additional management tactics to use alongside host resistance (Grabau 2022; Turner et al. 2023). Overall, reniform nematode-resistant cotton cultivars show potential to be a component of an integrated nematode management strategy in cotton production.
